# Molecular Analysis for Differential Diagnosis of Small Bowel Obstruction: Expression of Proinflammatory Cytokines and Diamine Oxidase Activity

**Published:** 2006-06

**Authors:** Teruyuki Akimoto, Moriatsu Takada, Takao Ichihara, Yoshikazu Kuroda

**Affiliations:** 1 *Department of Gastroenterological Surgery, Graduate School of Medicine, Kobe University, Kobe, Japan;*; 2 *Kobe Research Institute for Medical Sciences, Kobe, Japan*

**Keywords:** ileus, IL-6, TNF-α, IL-1β, diamine oxidase

## Abstract

**Background:**

A small bowel obstruction is classified as simple (nonstrangulated) or strangulated. The early recognition with correct diagnosis of small bowel obstruction is a critical issue as the release from strangulation requires surgical emergency.

**Methods:**

To evaluate the physiological effect on small bowel obstruction, a metallic ring was put in the small intestine (simple ileus) and a loop obstruction was made with keeping the blood flow (strangulated obstruction). Serum level of cytokines, IL-6, TNF-α, and IL-1β as well as endotoxin and seromuscular enzymes, CPK and LDH, were serially analyzed. Serum and mucosal DAO activity were also assessed.

**Results:**

Endotoxin was increased at 18 h through 48 h in strangulated obstruction, but not detected in the simple ileus. Early proinflammatory cytokines were significantly elevated in strangulated obstruction. High expression of IL-6 prolonged after 12h. Transiently expressed IL-1β peaked at 12h, TNF-α was increased at 18 h. In simple ileus, these expressions of cytokines were low and slow. LDH and CPK were significantly elevated at 48h, but there were no difference between simple ileus and strangulated obstruction. Serum DAO activity was significantly increased in simple ileus, but gradually decreased in strangulated obstruction, while mucosal DAO activity was decreased in both groups.

**Conclusions:**

High level of serum IL-6 is an early marker for strangulated obstruction. The pattern of serum DAO activity, decrease in strangulated obstruction and increase in simple ileus, might be useful molecular parameter in the early and proper diagnosis of small bowel obstruction.

## INTRODUCTION

A small bowel obstruction (SBO) can occur by various causes. SBO can be classified as simple (nonstrangulated) or strangulated. Nonstrangulated simple ileus (SI), manifesting the impairment of coordinated propulsive intestinal motility, remains a frequently documented and almost inevitable consequence of open abdominal surgery. Meanwhile, Strangulated obstruction (SO) is most commonly associated with adhesions and occur when a loop of distended bowel twists on its mesenteric pedicle. If it is not properly treated followed by early diagnosis, arterial occlusion leads to bowel ischemia, this progresses to perforation, peritonitis, and increases morbidity and mortality. If surgery is performed within 36 h, the mortality decreases to 8%. The mortality rate increases to 25% if the surgery is postponed over 36 h. As mortality and morbidity are dependent on the early recognition and correct diagnosis of obstruction, differential diagnosis is a critical issue as SO requires urgent surgical treatment. Some trials using CT to enable differentiation of SI and SO have been reported, but not sufficient for the diagnosis of SBO (1-3).

Despite the frequency and major impact of ileus on morbidity and mortality, the underlying molecular and cellular mechanisms of this important clinical conundrum have not been well defined (4). It has been reported that endotoxin and cytokines generated primarily by the gut, act as causative factors of sepsis-induced ileus (5). Most of the previous studies have merely been performed by administration of LPS or vessel ligation to induce ischemia, therefore, we have established to SBO models for SI and SO in order to investigate the actual effect of physiological obstruction. In this study, we have characterized the expression pattern of early proinflammatory cytokines, seromuscular enzymes and diamine oxidase (DAO) activity, trying to determine the useful molecular parameter for the early and proper diagnosis of SBO.

## MATERIALS AND METHODS

### Animals and Surgical Procedures

Inbred male Wister rats (12 weeks old, approximately 250-300 g of body weight) were obtained from Oriental Co. (Osaka, Japan) and kept under quarantine for one week before use in the experiments. Under anesthesia using secobarbital sodium (50 mg/kg intraperitoneal administration), animals were open-laparotomized with 4 cm incision at the midline of the abdomen and a tiny metallic ring (3 mm in diameter) was put in the small intestine near the ileum end (SI group). In SO group, the ring was put at the same portion of the U shaped loop obstruction of the small intestine (4 cm in length) with keeping the blood flow. Animals were from the same litter and five rats per group were used in each set of data points of the experiments that were repeated at least three times independently (n=15).

### Cytokines and diamine oxidase analysis

Blood samples (1 ml) for endotoxin determination were obtained from the tail vein seriously 0, 6, 12, 18, 24, 36 and 48 h. Endotoxin was measured by chromogenic endotoxin-specific assay using recombined limulus coagulation enzymes (limulus test, SRL, Tokyo, JAPAN). To identify specific cytokines, representative sera (5 rats/time point) were collected at 0, 6, 18, 24, 48 h. Production of IL-6, TNF-α and IL-1β were assessed by enzyme-linked immunosorbent assay (ELISA) using Biosource Cytoscreen rat ELISA kits (Fa. BioSource, Camarello, CA, USA). Serum DAO activity was measured based on a coupled reaction with peroxidase and a new chromogen, 10-(carboxymethyl-aminocarbonyl)- 3,7- bis (dimethylamino) phenothiazine sodium salt (6). Mucosal DAO activity was also analyzed after isolation from the intestinal mucosa followed by the methods as previously described (7).

### Statistical Analysis

The data are presented as arithmetic means ± standard deviation (SD). Statistical comparisons between groups were performed by Student t-test (unpaired, two-tailed). The difference was considered significant at *P*<0.05.

## RESULTS

### Serum level of endotoxin

We first evaluated the levels of endotoxin in SBO. Endotoxin was not detected in the SI group throughout the time period we examined. Although endotoxin was not detected until 12h in SO group, it started to increase at 18 h (2550.2 ± 321.9 pg/mL) through 48 h (2758.2 ± 296.5 pg/mL).

### Expression of early proinflammatory cytokines

**Figure 1:**
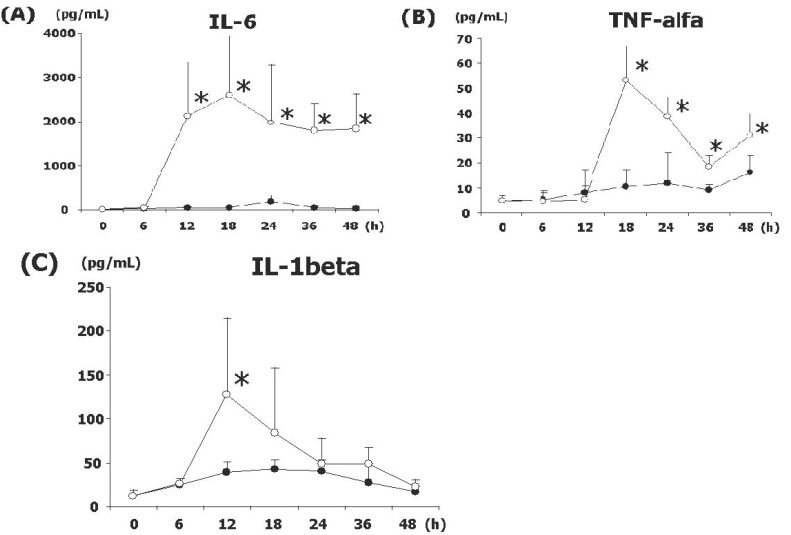
Expression of early inflammatory cytokine in SBO. Compared to the SI (closed), SO (open) shows significant increase of IL-6 (A), TNF-α (B) and IL-1β (C). Asterisks denote the statistical differences compared to SO (*P<0.05). N=15 for each sets of data points.

**Figure 2:**
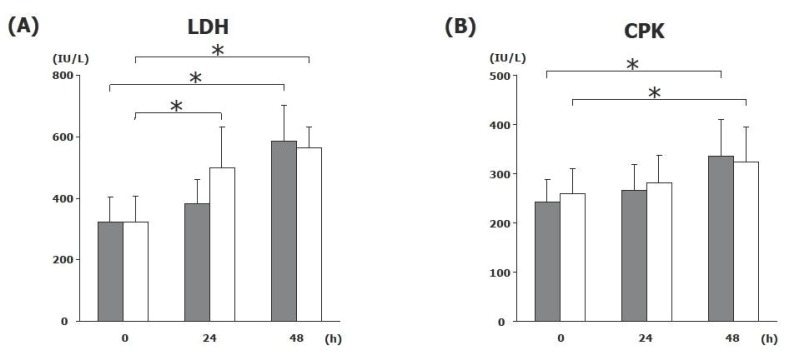
Serum level of seromusclar enzymes. Both SI (black), SO (white) show significant increase of LDH (A) and CPK (B). There were no differences between the groups. Significant differences are represented by asterisks (*P<0.05). N=15 for each sets of data points.

To determine the roll of early proinflammatory cytokines in SBO, the serum level of IL-6, TNF-α, and IL-1β were examined. As shown in Figure 1, all molecules we studied were significantly elevated in SO. IL-6, in particular, showed the prolonged expression after 12h at the level of more than 2000 pg/mL. IL-1β was transiently expressed, peaked at 12h, and later decreased to the basal level. TNF-α was increased at 18 h (53 pg/mL) and rapidly decreased, nevertheless, it started to increase at 48 h in both SI and SO. The degree of expression of these proinflammatory cytokines was low and slow in SI as shown in Figure 2.

### Delayed elevation of Seromuscular Enzymes

LDH is the commonly enzyme marker that indicates injury to the cells including muscle layer in the small intestine. Serum LDH activity was significantly elevated at 48 h, but there were no difference between SI and SO. Elevation of serum CPK level was seen after the muscle necrosis such as myocardial infarction and the injury of the muscle layer in the small intestine. Similar to LDH, the level of CPK was increased at 48 h, but there were no difference between the groups.

### Serum and mucosal DAO activity

Early marker for the mucosal changes, DAO activity, was assessed in the sera and the mucosa of the small intestine. While serum DAO activity was significantly increased in SI, it was gradually decreased in SO. Both SI and SO showed decreased level of mucosal DAO activity. Compared to the SI, mucosal DAO activity was rapidly decreased in SO.

## DISCUSSIONS

Endotoxin (lipopolysaccharide: LPS), a common component of the cell walls of gram-negative bacteria, exhibits various pathophysiological effects such as tissue injury and sometimes results in lethality (8). It has been reported that endotoxemia elicits an inflammatory response by activating of chemical mediators within the intestinal muscularis and causes gastrointestinal dysmotility (9). Although the effects of endotoxin have been well investigated by the injection of LPS, there is no distinct comparative study in the SBO models. Our models using a metallic ring physiologically induce SBO with keeping blood flow, which we can compare the effects of strangulation by itself. The previous methods of artificially clipping or ligation are the very ischemia/reperfusion effect that might be a different from the actual phenomenon in strangulation.

In the present study, we have identified endotoxemia in SO at 18 h and later on, but not in SI. Then, we have characterized the expression pattern of early proinflammatory cytokines. As level of all cytokines we examined significantly elevated in SO, the extent of inflammation might be more severe than SI. Particularly, remarkable increase of IL-6 was identified at 12h, which was earlier than the elevation of serum endotoxin, prolonged until the time of sacrifice. Transiently expressed IL-1β also peaked at 12h and returned to the basal level. Nevertheless, TNF-α was not increased at 12h, instead, peaked at 18 h when is the start time of endotoxemia. These results shown in the present study are very implicative because recent study using multiple organ failure rats demonstrates that TNF-α is elevated in both the intestinal tissue and plasma (10). In another report using intestinal epithelial cell line, TNF-α and histamine stimulation increased neutrophil adhesion to intestinal epithelial cells and this increased adhesion is inhibited by anti-CD11b and anti-CD18 monoclonal antibodies (15). It has been reported that the histamine and TNF-α released from activated the intestinal mucosal mast cells (IMMC) play an important role in the development of multiple organ failure (10). Our result that the delayed increase of TNF-α compared to IL-6 and IL-1β might be related to the different mechanism of cell infiltration during the strangulation of small intestine, indicating that the early activation of IMMC followed by the activation of neutrophils.

**Figure 3:**
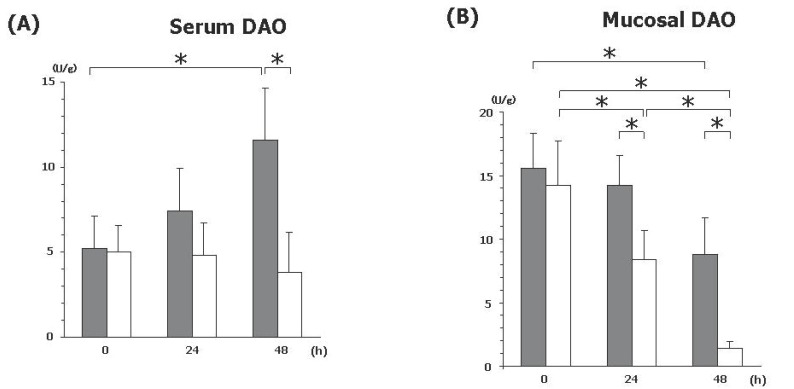
Level of DAO activity in sera and mucosa. (A) Serum DAO activity is gradually significantly increased in SI (black), however, it is decreased in SO (white). (B) Mucosal DAO activity is decreased in both groups. Significant differences are represented by asterisks (*P<0.05). N=15 for each sets of data points.

In this regards, we have analyzed early marker for the mucosal changes, DAO activity in the sera and the mucosa. DAO, histaminase, is a cytoplasmic enzyme found primarily in the villus epithelial cells of the small intestine and has a crucial role in the degradation of histamine in the small intestine (11, 12) (Figure 3). Previous report has shown that the changes in serum DAO activity indicate that the intestinal barrier is damaged after intestinal ischemia/reperfusion injury (13). Pathophysiological states that produce intestinal ischemia/reperfusion injury initiate an inflammatory cascade and result in ileus. Intestinal ischemia is associated with decreased intestinal DAO activity, which is influenced by the duration of intestinal ischemia (14).

Our result of the significant elevation of serum DAO activity in SI demonstrates the damaged intestinal barrier. As to mucosal DAO, they are gradually decreased because of the mucosal damage. The extent of decrease of mucosal DAO is more rapid in SO, indicating that the mucosal damage is more severe than SI. Interestingly, serum DAO activity is not increased but rather decreased in SO. This indicates that the reduction of blood flow due to strangulation might perturb the elevation of this enzyme in the blood. Thus, this might be the key molecular issue in the mechanism of strangulation. Clinically, the elevation of serum DAO activity (double to basal level) might be a useful marker for the diagnosis of SI to SO.

Gradual increase of CPK and LDH in both groups indicates the result of the intestinal wall damage caused by SBO. Unfortunately, none of these markers seem not to be beneficial for the early and differential diagnosis of SBO. Recent report using rat model has shown that increased serum hexosaminidase levels indicate irreversible transmural infarction only in the late period of strangulation in the closed loop SBO (15). Similar to hexosaminidase, DAO represents the biological activity in the mucosa, therefore, the present result of differential pattern of serum DAO activity might be a useful tool for diagnosis.

The high level of serum IL-6 might be an early useful marker if the clinical suspicion of SO arises, because elevation of IL-6 was identified at 12h and prolonged longer than other cytokine markers. Decrease of serum DAO activity also predicts SO, while the increase of serum DAO activity predicts SI. As the early DAO increase can be a marker to indicate the only SI, but a decrease of DAO in SO, this might be a possible clinical good marker for the differential diagnosis of these two SBO. Because strangulation requires the emergent surgery, these predictors might be useful tools for the differential diagnosis of ileus. Elevation of early proinflammatory cytokines, such as IL-6 and IL-1β, at the time of 12h might be an early implicative markers for SI, while TNF-α at 18h and serum DAO at 48h could be additional marker for the confirmation. As we have demonstrated, these approaches will be open the door to distinguish SI from SO. Further studies are needed to clarify the status of SBO since urgent treatment including surgery could reduce the risk of mortality and morbidity.
